# STEM Teacher Professional Learning Through Immersive STEM Learning Placements in Industry: a Systematic Literature Review

**DOI:** 10.1007/s41979-023-00089-7

**Published:** 2023-03-07

**Authors:** Mairéad Hurley, Deirdre Butler, Eilish McLoughlin

**Affiliations:** 1 Centre for Advancement of STEM Teaching and Learning & School of Physical Sciences, Dublin City University, Glasnevin, Dublin, Ireland; 2 School of Education, Trinity College Dublin, Dublin, Ireland; 3 Centre for Advancement of STEM Teaching and Learning & School of STEM Education, Innovation and Global Studies, DCU Institute of Education, Dublin City University, Drumcondra, Dublin, Ireland

**Keywords:** STEM education, Industry placements, Pre-service education, Professional learning, Professional development

## Abstract

Over the past two decades, there has been an increased focus on designing STEM learning experiences for primary and second-level students. We posit that for teachers to design rich learning experiences for their students, they must first have the opportunity to develop their own STEM knowledge and competences, either during their pre-service teacher education or as part of their professional learning as in-service teachers. This systematic review of literature examines programmes which offer either pre-service or in-service teachers immersive learning experiences through placements in STEM roles in business or industry. A total of nine papers were identified in this review, featuring three unique programmes—one in the UK for pre-service teachers, and two in the USA involving in-service teachers. The findings indicate a variation in motivation and structures across the three programmes. The influence on teachers’ personal and professional development, and their intentions to change their classroom practices or behaviours to incorporate more ‘real-world’ contexts into their STEM learning activities, inspired by their experiences in industry, is discussed. This study presents recommendations for the design and implementation of immersive learning placements in industry to support STEM teacher professional learning, as well as suggestions for further studies to examine the influence on their classroom practice.

## Introduction

As we prepare learners to live and work in a complex and connected society, it is becoming increasingly apparent that education should not be confined to single subject disciplines, and happen only within the school walls, but rather reflect the entangled and dispersed nature of 'real-world' challenges. STEM (Science, Technology, Engineering, Mathematics) education and STEM literacy have been increasing in prominence in scholarly research since the beginning of this century, as discussed by Li et al. (2020) in a review of trends in STEM education research between the years of 2000 and 2018. Likewise, STEM education has been widely adopted in educational policy worldwide (Anderson, 2020), although as noted by Freeman et al. (2014) the ‘discipline grouping, and the term itself, are not used uniformly in international educational policy or practice’ (p. 3).

### Towards Integrated STEM Education

The purpose of STEM education in schools has been challenged by various scholars. Weinstein et al. (2016) argue that the STEM discourse can ‘reinforce and legitimize a neoliberal hegemony of global competition and capitalist expansionism’ (p. 201). Hallström and Schönborn (2019) posit that ‘authenticity must be viewed as a cornerstone of STEM literacy’ (p. 1), while Pitt (2009) and Williams (2011) caution that authentic STEM education should push against the so-called STEM crisis discourse and shift the focus away from the economic notion of STEM in service of international competitiveness towards one of an interdisciplinary way of learning. Andrée and Hansson (2020) also discuss the problematic ‘STEM crisis’ discourse and suggest that it is fuelled by frequently used terms like ‘leaky talent pipeline’, and the narrative of STEM progress with respect to competition between nations for economic advantage. Moving beyond solely focusing on the economic imperative, European Schoolnet (Nistor et al., 2018, p.14) suggests that the aims of promoting STEM initiatives are to:

- popularise sciences (increase STEM literacy),
- increase STEM uptake by promoting STEM careers,
- engage the gifted and talented with challenging STEM initiatives,
- reduce the gender gap in STEM.

Consensus on the definition of the term ‘STEM education’ has still not been reached within academic spheres (Bybee, 2013; McLoughlin et al., 2020). Indeed, many issues remain unresolved—for example, implementation of a coherent approach to STEM education continues to be vague (Granshaw, 2016). Moore and Smith (2014) also emphasise the importance of authentic contexts in their definition of STEM education, highlighting the need for ‘an effort to combine some or all of the four disciplines of science, technology, engineering and mathematics into one class, unit or lesson that is based on connections between the subjects and real-world problems’ (p. 5). Martín-Páez et al. (2019) states that ‘using real-world examples improves STEM learning’ (p. 813) and suggests that an authentic context is crucial when designing and implementing a STEM‐focused educational intervention. However, research continues to point to the failure of educational systems in supporting students to understand how to solve real-world problems using knowledge gained through STEM disciplines (Bybee, 2013; Ritz & Fan, 2015). Such observations may be attributed to the lack of a clear understanding of what is understood by STEM education in practice.

In broad-ranging reviews spanning the burgeoning field of STEM educational research, Li and colleagues caution that while STEM education offers new opportunities for teaching and learning, there is no widespread consensus on the limits of what this compound discipline should encompass (Li & Xiao, 2022; Li et al., 2020, 2022). Several authors have carried out reviews of the various definitions and understandings of STEM education and have found that they span a range of levels of integration across and between the individual subject disciplines, from educational approaches which combine any number of the four disciplines, to those which integrate all four disciplines in an authentic, real-world context (Bybee, 2013; Martín-Páez et al., 2019; McLoughlin et al., 2020; Thomas & Watters, 2015). A central theme in integrated STEM education research (e.g. Martín-Páez et al., 2019; McLoughlin et al., 2020) is the value of using real-world contexts as a basis for designing ‘authentic’ STEM learning opportunities in the classroom. However, connecting ideas across disciplines is challenging when students have little or no understanding of the relevant ideas in the individual disciplines. A systematic literature review by McLoughlin et al. (2020) identified seven characteristics of integrated STEM education: (1) Key STEM Competences^1^; (2) Problem Solving Design and Approaches; (3) Disciplinary and Interdisciplinary Knowledge; (4) Engineering Design and Practices; (5) Appropriate Use and Application of Technology; (6) Real World Contexts; and (7) Appropriate Pedagogical Practices. Designing integrated learning experiences providing intentional and explicit support for students is important to build knowledge and competences both within and across disciplines. The use of appropriate resources (including use of digital tools and resources), innovative pedagogies and curriculum innovation, are particularly important in facilitating an integrated approach to STEM education. Teachers can be supported with resources and introduced to innovative pedagogies through appropriate professional learning opportunities (Butler et al., 2020).

In their systematic review of the literature relating to teachers’ perceptions of STEM integration and education, Margot and Kettler (2019) state that ‘the pedagogical strategies associated with STEM must be explicitly taught to teachers and modelled in order to improve fidelity of programming’ (p. 14). Butler et al. (2020) highlight the need for teachers to be engaged in professional learning experiences and collaborative opportunities across disciplines to enable them to identify crosscutting knowledge and/or skills, and become familiar with the current practices of an interdisciplinary STEM curriculum. Ryu et al. (2019) also highlight the importance of role models for teachers themselves, many of whom may not have been introduced to integrated STEM learning within their own school experiences. To support teachers in designing integrated STEM learning opportunities, teacher educators need to model appropriate pedagogical practices that integrate STEM disciplines in the programmes they design and facilitate for teacher education. In addition, providing teachers with opportunities to participate and engage in professional learning communities with experienced STEM teachers is beneficial. However, as there are limited examples of appropriate tools and strategies that can be used by teachers in the STEM classroom, this highlights the need to support teachers’ in developing their own pedagogical and assessment practices for designing integrated STEM learning (Butler et al., 2020).

### Teacher Professional Learning

Teacher professional placements during initial teacher education are often limited to school-based placements in which pre-service teachers observe practising teachers and begin to develop their own classroom practice (Allen & Wright, 2014; Darling-Hammond, 2006). Some teacher education programmes offer ‘alternative’ practicum placements for teachers, which introduce teachers to workplaces other than the classroom (Larsen & Searle, 2017; Purdy & Gibson, 2008), to ameliorate the case that it is entirely possible for a young person to move from the role of school student to university student, and then back into a school as a teacher, without experiencing any other working environments. A crucial role for teachers is to support their students as they consider the range of future learning and career pathways they may choose to explore. However, STEM subjects, as studied in the school or even university classroom, very rarely mirror the everyday practices in the range of roles related to STEM. Kyndt et al. (2021) propose a rethinking of the traditional boundaries between education and employment, stating: ‘Future-oriented education needs to invest in the connectivity between learning and working in order to realise its full potential’ (p.i).

A significant body of research exists in relation to teacher professional learning (e.g. Darling-Hammond et al., 2017; Guskey, 2002) and development of teachers’ pedagogical content knowledge (Shulman, 1986; Uzzo et al., 2018). However, there is limited focus on professional learning opportunities for teachers in industry or other non-school based placements (Perry & Ball, 1998; Zaid & Champy-Remoussenard, 2015). According to White et al. (2018), school-industry engagements offer increased authentic learning opportunities, and can enable school-to-work transitions. Margot and Kettler (2019) emphasise the demand for extended professional learning opportunities to develop teacher confidence and competence with STEM education, stating that ‘the most often mentioned support that would increase the effectiveness of STEM education was learning opportunities for teachers to increase their ability to effectively integrate STEM content into their curriculum’ (p. 14).

According to ‘situated learning theory’ (Lave & Wenger, 1991), learning happens through legitimate participation in real-world activity, rather than out of context. Collaboration is key and learning progresses through social interactions with experienced peers, and apprenticeships as a member of a community of professional practice. Teachers may better prepare students for the future by enriching their own education through participation in situated learning opportunities, and developing relevant skills within the workplace context. Holvoet and Wante (2021) discuss situated learning for teachers through practical experience in workplaces relevant to the subjects that they teach, and state that to ‘be able to create authentic learning environments in education, teachers need to obtain relevant and up-to-date experiences in the workplace’ (p. 51). They present a study of five higher education teachers who undertook a month-long placement (called an externship) in an industry setting related to their teaching discipline—these included healthcare, business, education, psychology and engineering (Holvoet & Wante, 2021). The research design probed the hopes and aspirations of the participating teachers in advance of the externship. The teachers indicated that they hoped to expand their content knowledge about industry practices, as well as their pedagogical content knowledge. The findings from a post-placement follow up indicate that as a result of participating in these externships, these goals were met in most cases, and several participating teachers also reported positive personal outcomes such as an increased passion for teaching or increased self-confidence. However, the authors (Holvoet & Wante, 2021) caution that there are limited research findings that corroborate the relationship between the design of a workplace immersion experience for teachers and associated teacher learning. A potentially useful tool for teachers in translating their industry experiences into authentic real-world classroom learning experiences is the concept of ‘Industrial Content Knowledge’ (Finlayson et al., 2020), that supports teachers in using real-world industrial contexts in their inquiry-based science lessons.

### Teacher Placements in STEM Roles in Industry

The means to support teachers, and hence learners, to make connections between STEM disciplines in the classroom could be facilitated by enabling teachers to personally experience STEM roles in the workplace. Such opportunities could support teachers in designing STEM teaching and learning activities that are aligned with school/subject curricula and promote STEM learning experiences rooted in real-world contexts. Several innovative programmes are emerging worldwide, which offer teachers immersive experiences in STEM industry or research settings, enabling them to gain first-hand experience of ‘real-world’, or authentic STEM contexts that they could then translate into appropriate learning experiences for their students. Two of the authors of this review (Butler & McLoughlin) lead such a programme in Ireland that aims to provide immersive STEM learning experiences for pre-service primary and secondary teachers through a 12-week paid internship in a STEM role in industry (Hurley et al., 2021). The ambition to inform the design of this programme in line with international examples, and to capture the impact of the programme through rigorous research and evaluation, has provided the impetus for this review. The authors posit that internships in industry can offer teachers unparalleled access to authentic, real-world STEM contexts, that can deepen and/or extend their content knowledge and awareness of STEM subjects, careers, and diverse STEM role models. The authors believe that these internship experiences provide invaluable support for pre-service and early-career teachers in designing integrated STEM learning experiences for their students in the classroom.

Given the perceived importance of ‘real-world’ contexts in integrated STEM education (McLoughlin et al., 2020), and the need for teacher professional learning opportunities for STEM education (Margot & Kettler, 2019), the aim of this systematic review is to examine existing literature to determine the prevalence, objectives, and outcomes of programmes in which pre-service or in-service teachers spend an extended period on a STEM-related industry placement. The term ‘industry’ is used to refer to organisations that are distinct from higher education, and in general are for-profit entities. This review concerns teachers developing their understanding of STEM roles, either within traditional STEM industries such as technology, engineering or pharmaceutical sectors or industries which have STEM-related roles (e.g. within consulting firms).

This study aims to examine the opportunities that exist internationally for teachers to complete a placement programme in industry, the experiences of these participating teachers and the influences on their classroom practices. Based on these aims, the research questions of this systematic review are:

1. What are the purposes and objectives of STEM-related teacher placement programmes in industry?
2. Does the experience of undertaking placements in STEM-related workplaces influence teachers’ behaviours and intentions towards STEM education?

## Methods

This systematic literature review attempts to answer the research questions outlined above, following the steps used by Goos et al. (2020) for a systematic review on a related topic. A web-based tool named Covidence (www.covidence.org), which supports systematic literature reviewing, was utilised. Covidence allows researchers to import and screen citations, titles and abstracts as well as full-text articles, and to extract data using customisable forms. Covidence tracks decisions made by multiple reviewers and has a function to track conflicts until the review team resolves them and allows the review team to export results in various formats.

### Data Sources and Searching

The protocol for specifying inclusion/exclusion criteria and eligibility and the search strategy was defined by the researchers in collaboration with the Dublin City University Institute of Education specialist subject librarian. The following electronic databases covering the areas of education and social science were searched: EBSCO Education Research Complete, Scopus, ERIC International (which covers ERIC and the Australian Education Index), Taylor and Francis, Wiley and Web of Science. All searches were initially carried out in April–May 2022 and updated in September 2022. All searches were limited to those articles published after 1st January 1998, as the term ‘STEM’ entered the educational lexicon after this date. Articles were searched by title, abstract and keywords, and limited to English language publications. The search terms were refined initially in EBSCO Education Research Complete using the thesaurus function, and keyword searching was used in all databases, except for Taylor & Francis, in which the term ‘world of work’ was removed as it resulted in too many unrelated hits. The searches in the Wiley and the Taylor and Francis databases were limited to journals classified as ‘education’. The results of the initial search are presented in Table 1.

**Table 1:** Results of searches conducted on six databases

Search terms	Database	Search limiters	Hits
(‘teacher education’ OR ‘teacher training’ OR ‘teacher preparation’ OR ‘pre?service teacher’ OR ‘student teacher’ OR ‘teacher development’ OR ‘professional learning’ OR ‘professional development’) AND (‘industry engagement’ OR ‘industr* placement*’ OR ‘industr* internship*’ OR ‘teacher externship*’ OR ‘academic-industrial collaboration’ OR ‘industr* secondment’ OR ‘education-industry partnership’ OR ‘world of work’ OR ‘educator externship’ OR ‘teacher internship’ OR ‘educator internship’)	EBSCO Education Research Complete	Since 1998	165
(‘teacher education’ OR ‘teacher training’ OR ‘teacher preparation’ OR ‘pre?service teacher’ OR ‘student teacher’ OR ‘teacher development’ OR ‘professional learning’ OR ‘professional development’) AND (‘industry engagement’ OR ‘industr* placement*’ OR ‘industr* internship*’ OR ‘teacher externship*’ OR ‘academic-industrial collaboration’ OR ‘industr* secondment’ OR ‘education-industry partnership’ OR ‘world of work’ OR ‘educator externship’ OR ‘teacher internship’ OR ‘educator internship’)	Scopus	Title, abstract or keyword search; Since 1998	123
(‘teacher education’ OR ‘teacher training’ OR ‘teacher preparation’ OR ‘pre?service teacher’ OR ‘student teacher’ OR ‘teacher development’ OR ‘professional learning’ OR ‘professional development’) AND (‘industry engagement’ OR ‘industr* placement*’ OR ‘industr* internship*’ OR ‘teacher externship*’ OR ‘academic-industrial collaboration’ OR ‘industr* secondment’ OR ‘education-industry partnership’ OR ‘world of work’ OR ‘educator externship’ OR ‘teacher internship’ OR ‘educator internship’)	ERIC International	Since 1998	140
[‘teacher education’ OR ‘teacher training’ OR ‘teacher preparation’ OR ‘pre?service teacher’ OR ‘student teacher’ OR ‘teacher development’ OR ‘professional learning’ OR ‘professional development’] AND [‘industry engagement’ OR ‘industr* placement*’ OR ‘industr* internship*’ OR ‘teacher externship*’ OR ‘academic-industrial collaboration’ OR ‘industr* secondment’ OR ‘education-industry partnership’ OR ‘educator externship’]	Taylor and Francis	Subject: education; Since 1998	63
‘teacher education’ OR ‘teacher training’ OR ‘teacher preparation’ OR ‘pre?service teacher’ OR ‘student teacher’ OR ‘teacher development’ OR ‘professional learning’ OR ‘professional development’ AND ‘industry engagement’ OR ‘industry placement’ OR ‘industry internship’ OR ‘teacher externship’ OR ‘academic?industrial collaboration’ OR ‘industry secondment’ OR ‘education?industry partnership’ OR ‘educator externship’	Wiley	Subject: education; Since 1998	18
(‘teacher education’ OR ‘teacher training’ OR ‘teacher preparation’ OR ‘pre?service teacher’ OR ‘student teacher’ OR ‘teacher development’ OR ‘professional learning’ OR ‘professional development’) AND (‘industry engagement’ OR ‘industr* placement*’ OR ‘industr* internship*’ OR ‘teacher externship*’ OR ‘academic-industrial collaboration’ OR ‘industr* secondment’ OR ‘education-industry partnership’ OR ‘educator externship’)	Web of Science	Since 1998	18
**Total after removal of duplicates, including 9 items selected from Google Scholar, and 6 items discovered via hand-searching**			**423**

A search was also conducted using Google Scholar for the same period. While Google Scholar does not have the same advanced search capabilities as the other databases, the search string ‘*teacher*’ ‘*STEM*’ *industry OR or OR externship OR or OR internship* was used (i.e. with **all** of the words ‘teacher’ and ‘STEM’, and with at least one of the words ‘industry’ or ‘externship’ or ‘internship’). Following the suggestion of Haddaway et al. (2015), the first ~ 200 titles in the Google Scholar results were screened to identify any potential omissions. Nine potentially relevant publications were located which had not shown up in the other database searches, and these were selected and imported into Covidence for title and abstract screening.

A hand search was also conducted following the full text-screening phase, examining the literature cited in the publications that were retrieved. The researchers also engaged in private communication with an author whose publication was identified as relevant during the full-text screening stage, to discuss whether there were any identifiable gaps in the search. These additional methods resulted in the discovery of six further publications.

### Study Selection

This review focussed on pre-service or in-service teachers of STEM subjects at either primary or secondary level (students aged 5–18 years approximately). Studies only referring to pre-school (i.e. 0–5 years), higher or vocational education were excluded. Studies that reported on teacher placements in business or industry of less than 20 hours were excluded, as this was deemed to be the minimal duration for effective teacher professional development (Guskey & Yoon, 2009; Yoon et al., 2007). Studies that did not involve in industry placement were excluded. Following the removal of duplicates, the initial screening process involved reviewing the title and abstract of the remaining 423 articles against these eligibility criteria. Following this initial screening, 33 articles were retained for full-text review. Articles were retained for full-text review if it was not clear from the title and abstract whether any exclusion criterion had been met.

**Fig. 1:**
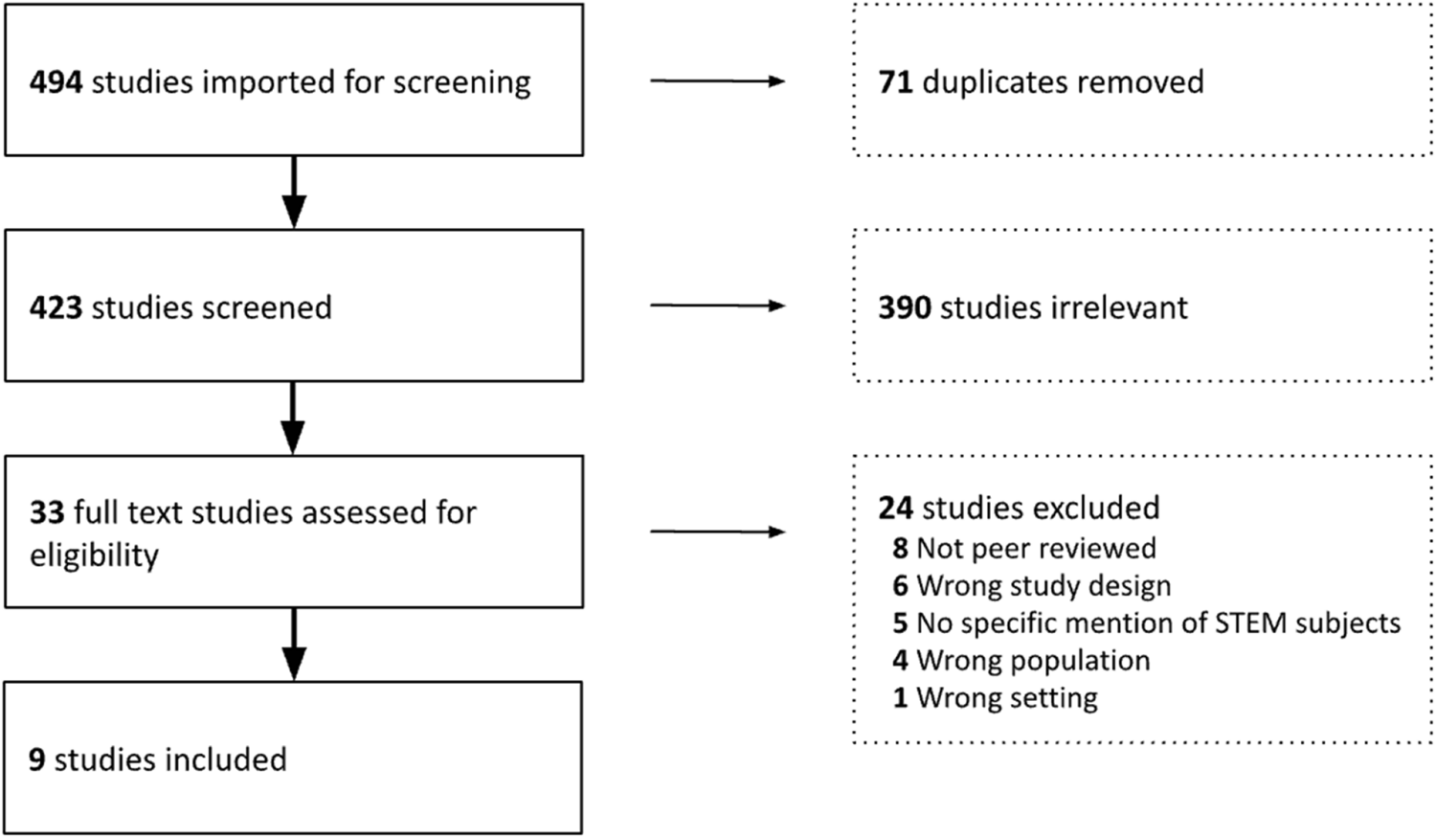
PRISMA flow chart for the screening process, showing the number of articles removed at each stage of the systematic literature review process, leading to nine included studies

After full text review of the retained articles, an additional twenty-four articles were deemed to be excluded (exclusion criteria indicated in Fig. 1), leaving nine in the final sample, of which six are conference proceedings and three are journal articles, as shown in Fig. 1. Studies that referred to placements in research or academia only were excluded, but studies in which placements were hosted in either industry or research/academia were retained. Articles were also excluded if they were not peer reviewed, or had no specific mention of STEM disciplines.

### Evaluation and Data Extraction

A quality assessment rubric was used to evaluate the suitability of the included literature, adapted from Margot and Kettler (2019). The nine studies met or exceeded standards in all five criterion categories: objectives and purposes, participants, methods, results and conclusions and significance.

From the nine selected articles, the following information was extracted: (a) country, (b) programme name and location, (c) placement duration, (d) participant information, e.g. primary/secondary teacher or grades taught, subjects taught, pre-service/in-service, (e) programme eligibility criteria, e.g. minimum number of years teaching experience, (f) programme funding sources, (g) study design and methodology, (h) number of participants and (i) key findings. These findings are summarised in Table 2.

**Table 2:** Overview of studies referring to STEM industry placements for teachers

Programme ID	GIFT program	Educators in industry: K-12 externships	Student placement, B.Ed., technology and design, Stranmillis University College
Location	USA: Georgia	USA: North Dakota	UK: Northern Ireland
Duration	7–8 weeks	4 weeks	5 days
Teacher career stage	In-service	In-service	Pre-service
Number of teachers involved in study	152 Breakdown: 50 (Barrett & Usselman, 2005) 102 (Barrett & Usselman, 2006)	101 Breakdown: 5 (Bowen, 2013); 10 (Bowen, 2014); 18 (Bowen, 2015); 33 (Bowen, 2016); 9 (Bowen & Shume, 2018); 26 (Bowen & Shume, 2020)	10
Accreditation/Qualification obtained	Yes—up to 10 professional learning unit	Yes—up to 3 continuing education credits	Yes—accredited module in a degree course for pre-service secondary / post-primary teachers of Design & Technology (combined subject in UK)
Subjects & grade levels	K-12 science, mathematics & technology teachers Grades taught were: High school (58%), middle grades (38%), Primary (4%)	Grades 3—12 STEM teachers and school educators such as librarians or guidance counsellors	Secondary / post-primary teachers of Design & Technology (combined subject in UK)
Key findings	Over 70% of respondent teachers: - Experienced real-world applications of STEM and got to use new equipment - Gained a greater understanding of the applications of science, mathematics, and/or technology in everyday life - Increased their knowledge of current issues in scientific or mathematical research - Increased their knowledge of careers that utilise science, mathematics, and/or technology - Now place increased emphasis in their teaching on cross-curricular, inquiry and hands-on activities - Increased their ability to incorporate ‘real life’ examples of the subjects they teach - Reported increased confidence levels and enthusiasm for teaching and learning	The influence of the programme on participating teachers, categorised into five key themes: (a) value of problem-solving, (b) importance of collaboration, (c) importance of communication, (d) using ‘real-world’ connections, and (e) casting students as employees. After participation, teachers had: - Changed perceptions about the importance of using the engineering design process and STEM in class - Increased understanding of twenty-first century skills and how they are used in industry - Changed perceptions about the importance of incorporating twenty-first century skills into classroom activities and planned to increase the frequency of student-led discussions and presentations and asking students to critique their work - Changed perceptions about the importance of interdisciplinary collaboration and were more likely to seek out opportunities for professional development in this area - Increased commitment to applying real-world examples and hands-on learning into their lessons - Actively encouraged students to explore alternative problem-solving methods - Draw upon externship experiences to foster authenticity and workplace readiness in their classrooms	The learning gains, both personal and professional, noted by participants were categorised by: - Activities undertaken: Touring the factories and conversing with employees. Students noted the linkage between technology, design, and practice - Student enjoyment: observing the factory and understanding the processes to transform the initial material to finished product - Student learning: Technical and procedural knowledge, the value of communication, disruption of gender-based stereotypes of engineers - Benefits and the relevance for the student: More ‘real world’ knowledge to share in classrooms - Professional benefits: Network connections, tangible experiences to build into lesson plans - The curriculum: Students linked their experiences to the curriculum - Technology & Design: Greater understanding of practice and application of this field - STEM links: Application of STEM theories into the ‘real world’ was observed through placements - Learning for life and work: Better understanding of life outside of education

### Data Analysis

The literature was analysed following the thematic analysis approach of Braun and Clarke (2006), to identify repeated patterns of meaning. An inductive or bottom-up approach was primarily adopted, reading the literature and assigning codes to segments of the texts rather than imposing codes based on existing theoretical or conceptual frameworks. However, we did search for specific instances of ‘core STEM competences’ (McLoughlin et al., 2020) and coded for these: collaboration, problem-solving, innovation and creativity, critical thinking, disciplinary skills and competences, self-regulation, communication and metacognitive skills.

### Limitations

The aim of this paper was to explore findings from literature on the opportunities and impact of industry-based professional learning for primary and second-level teachers of STEM subjects. Hence, the focus of the study was limited specifically to teacher professional learning interventions within industry settings. Within this relatively small subfield, our search was refined further to examine only those interventions within STEM industries. Furthermore, studies that examined placements of less than 20 hours, or placements involving higher-education or vocational teachers, were also excluded. The use of this stringent search criteria means that the relatively small amount of literature uncovered amounts to evidence from only three unique programmes in total. Due to the small sample size, generalisations cannot be inferred about the merit of this approach to teacher education or professional learning. However, it also indicates the dearth of peer-reviewed research relating to this approach, and this study may serve as a starting point for further development and research in this area.

## Findings

Table 2 presents an overview of the key findings of these nine articles, which are related to three distinct programmes. One of the retained articles reported on a 5-day placement experience for pre-service teachers in Northern Ireland, UK (Gibson, 2013). Two articles refer to the Georgia Intern Fellowships for Teachers (*GIFT*) program. *GIFT*, offered to in-service teachers, has a 7–8-week duration. Six articles from 2013 to 2020 refer to the evolution of the programme Teachers in Industry: K-12 Teacher Internships in North Dakota, which had a duration of 4 weeks. This was initially only available to in-service teachers, but later was extended to include other school educators such as librarians or guidance counsellors, at which point the programme was rebranded ‘Educators in Industry: K-12 Externships’. It is not clear from the literature what prompted the change from the term ‘internships’ to ‘externships’ or what the authors consider the difference between the two.

An initial set of codes was developed from reviewing the nine selected articles. These were refined into three themes, discussed in the following sections:

- Programme design—motivation and implementation
- Teacher personal and professional learning
- Teacher behaviours and intentions.

### Programme Design

This theme relates to the motivation to design an industry placement programme for STEM teachers, as well as the programme design and operation. Generally, this theme relates to decisions made by programme directors in universities and teacher-education institutions.

#### Programme Design—Motivation

The three distinct programmes described in the included literature—one in the UK and two in the USA—are all led by programme directors from universities or teacher education institutions. The programmes claim to respond to national STEM education policies or standards, which are based on economic demands for more scientifically literate citizens, and the need for more people to complete STEM qualifications and a more qualified STEM workforce. All three programmes are based on the premise that offering teachers the opportunity to experience STEM industries first-hand will lead to improved STEM teaching and learning in the classroom, and hence increased student attainment and interest in STEM subjects.

In the UK programme (Gibson, 2013), the industry placement described is part of an initial teacher education course for secondary-school teachers of design and technology, while the remainder of the articles reviewed refer to two programmes in the USA which are aimed at in-service teachers and educators. Interestingly, the programme discussed by Bowen and Shume (2018, 2020) caters for STEM teachers as well as non-classroom educators, such as librarians, career guidance counsellors and administrators. ‘Educators in Industry’ (Bowen & Shume, 2018, 2020) has been in existence in North Dakota since 2011 and is reported as being ‘a collaborative effort of different entities including university faculty, local economic development corporations, education cooperatives, and businesses’ (p. 6). The other US initiative included in this review is the *GIFT* programme (Barrett & Usselman, 2005, 2006). These authors mention having an Advisory Board composed of industry leaders; however, there is no specific information in these articles about the initial development of the programme, or whether it was devised in collaboration or consultation with industry partners, or in response to the needs of industry.

With regards to the motivation for the design of these programmes, several of the articles refer to teachers’ lack of experience in relation to STEM and suggest that industry immersion experiences address this gap. Within the small sample of student teachers studied by Gibson (2013), ‘all the students concerned started their teacher education degree straight from school and therefore are without industrial experience.’ (p. 289). Bowen and Shume (2018) recognise the challenge for teachers to create authentic learning environments in the classroom without having any knowledge of how different processes and technologies are used in industry to solve problems. A key focus of the ‘Educators in Industry’ programme (Bowen & Shume, 2018, 2020) is to support teachers in developing their understanding of the engineering design process as a problem-solving technique that they can use in the classroom. The programme administrators collaborate with the host organisations during the placement planning stage to ensure that teachers experience as many different steps of the engineering design process as possible. Teachers observe projects in different stages of development, which supports their understanding of the work environment, the associated career opportunities, and the skills required for such roles.

A further theme identified as a motivation for offering industry experiences to teachers is towards preparing the next generation of learners for STEM careers. Bowen and Shume (2020) highlight the needs of STEM industries in terms of future employees, and the need for these workers to possess ‘workforce readiness’ skills, including ‘problem-solving, teamwork, adaptability, innovation, leadership, time management and design-thinking’ (p. 75). Bowen and Shume (2020) also suggest that ‘twenty-first century skills’—collaboration, creativity, critical thinking and communication—are important for highly effective employees. These authors claim that immersion programmes in industry ‘provide valuable professional learning experiences that show teachers first-hand the skills needed to solve current technological challenges, and impact teachers’ perceptions of the need to integrate workforce readiness skills into classroom activities’ (p. 75). The authors propose that this positions their programme as serving national economic goals in terms of the STEM workforce and competitiveness.

Barrett and Usselman (2006) also state that a motivation for the *GIFT* programme was for corporate partners to gain a better understanding of education. Within the small case study presented by Gibson (2013), there are references to a number of challenges that arose due to the lack of three-way communication between universities, industry and education. This theme also emerges within the *GIFT* and *Educators in Industry* programmes, indicating that this is a crucial factor if a programme is to be successful in bridging the gap between education and industry. The university as the programme facilitator has a key role to play in translating the ‘lack of understanding or perhaps maybe more accurately a total misconception of what each, education and industry, does and why’ (Gibson, 2013, p. 295).

Of the nine articles reviewed, only Barrett and Usselman (2006), referring to the *GIFT* programme, articulated a desire for the experience to be personally rewarding for the teachers, with the aim ‘that teachers return to their classrooms enriched and rejuvenated following their summer experience’ (p. 11.247.9).

#### Programme Design—Implementation

In each of the three programmes, the individual context influenced design and implementation in terms of duration, host organisations, programme elements and programme accreditation.

- Duration

Placement durations varied from 5 days (Gibson, 2013) to 4 weeks (Bowen & Shume, 2018, 2020), with the longest being the *GIFT* programme (Barrett & Usselman, 2005, 2006) with an average duration of 7–8 weeks. The duration of the programmes greatly influences the scope of the professional learning possible, ranging from a primarily observational experience (Gibson, 2013) to a more immersive workplace experience (Barrett & Usselman, 2005, 2006).

- Host type

The UK programme described by Gibson (2013) serves pre-service teachers of design and technology, and hence has more industry partners active in those industry sectors, particularly manufacturing. The ‘Educators in Industry’ programme described by Bowen and Shume (2020) has a primary aim to support teachers to ‘gain an understanding about the kinds of knowledge, skills and dispositions essential for successful employment in engineering-based work environments’ (p. 74), and hence focuses more on engineering-related roles and projects. The *GIFT* programme (Barrett & Usselman, 2006) covers both industry and academia/research placements for teachers. In this review, we chose to limit our search to programmes that offer industry-related work experiences for teachers and exclude those dealing only with research experiences for teachers (RETs). The *GIFT* programme was retained as it does include industry placements as well as RETs. There is a significant body of literature available on RETs (e.g. Miranda & Damico, 2013; Pop et al., 2010), but as discussed by Barrett and Usselman (2005), there are considerable differences between placements in industry or corporate settings and research environments. These ‘differences primarily include exposure to two different ‘worlds’—the world of a major corporation with thousands of employees generating products or a service, striving to maintain efficiency and excellence while delivering a sound product, or the much smaller, but highly intense world of the academic research laboratory’ (p. 10.608.5).

- Programme Elements

The articles reviewed provided an extensive overview of the types of placement activities in the three programmes. This includes examples of projects developed by teachers on placements (Barrett & Usselman, 2005; Bowen, 2013), as well as activities designed to enhance the transfer of the learning from the industry site to the classroom.

A common feature across all three programmes was the inclusion of orientation and other meetings with programme administrators to support teachers’ completion of the programme. As part of the *GIFT* programme experience, each teacher meets weekly in a subgroup of 10–15 teachers with a ‘master-teacher facilitator’ to develop an action plan to transfer their learning back to the classroom. Teachers also receive follow up and support from staff during the following academic year to implement their action plan.

Within the ‘Educators in Industry’ programme, the teachers on placement work in their industry host from Monday to Thursday, with a half-day on Fridays dedicated to professional development with the other participating teachers and the programme team (Bowen & Shume, 2018, 2020). Gibson (2013) describes university staff visits to the placement sites, in which they observe the work setting and conduct meetings with participating teachers and their industry mentors.

- Reimbursement

The participating in-service teachers in the ‘Educators in Industry’ programme receive a stipend for their time—Bowen (2013) reports this as $2000, ‘half of which is paid by the company and the other half is paid for by the local regional education association’ (p. 23.1134.4). In a later article (Bowen & Shume, 2018), no amount is mentioned for the stipend, but half was reported as paid by the ‘local Economic Development Corporation’ (p. 7). Participants in the GIFT programme receive a $5000 stipend for their 7-week placement, but no information is provided on the source of this funding (Barrett & Usselman, 2006). Pre-service teachers participating in the UK study are not paid for their industry experience—this small-scale programme was run at ‘no-cost’ (p. 297). Gibson (2013) accredits the success of the programme to being largely dependent on the willingness of cooperating host companies.

- Accreditation

Gibson (2013) describes pre-service teachers carrying out their 5-day industry placement as a requirement of an undergraduate module in their initial teacher education programme. The students were required to submit teaching and learning materials to receive the course credits for this module. Participating teachers in the ‘Educators in Industry’ programme also receive continuing education credits by enrolling in summer and fall courses at the coordinating university. The teachers are required to develop lesson plans during the summer as part of their ongoing professional learning seminars. They are also required to implement these lessons in the fall. The credits accrued by the teacher can be used towards the renewal of their teaching license. Teachers participating in the GIFT programme may obtain up to 10 professional learning units upon completion of programme requirements; however, information is not provided on what these credits may be used for by the participating teachers.

### Teacher Personal and Professional Learning

This theme relates to the personal and professional learning of the participant teachers themselves, on short and longer timescales. Generally, this section refers to self-reported outcomes from teachers, such as changes in knowledge, attitudes, behaviours, professional values or teaching styles. Professional learning was discussed in each of the nine articles reviewed. Teachers across the three programmes reported increased adaptability, confidence levels and enthusiasm for teaching, improved technology skills and deepened knowledge of the processes and practices used in real-world applications of STEM. For many of the participating teachers, it was extremely beneficial for their own learning to gain first-hand experience of the processes involved in STEM industries, from design to final product, especially within engineering or manufacturing sectors. Gibson (2013) reports the pre-service teachers frequently made links between the processes and tools used in the industry settings, and the scaled-down versions of the same that are used in the design and technology classroom.

The *GIFT* programme places an emphasis on the development of a professional learning community (Barrett & Usselman, 2005). This programme has been running since 1991 and reports to have had over 600 alumni by 2006. The programme website reports that it continues to run and by 2021 had placed teachers in over 2000 positions across the state of Georgia.^2^ Both Gibson (2013) and Barrett and Usselman (2005) highlight the benefit for teachers in developing their own personal connections and networks with industry mentors, emphasising that these may be useful for planning future school visits to industry, inviting industry representatives into the classroom or to leverage these connections for resources or other teaching materials in the future.

#### Core STEM Competences

A central element within this theme is the development of teacher understanding of, and appreciation for, the transversal skills and competences required for a career in STEM, often referred to as ‘twenty-first century skills’. We adopt the term ‘core STEM competences’ which incorporates twenty-first century skills alongside other key competences required in STEM education, namely collaboration, problem-solving, innovation and creativity, critical thinking, disciplinary skills and competences, self-regulation, communication and metacognitive skills (McLoughlin et al., 2020).

Bowen and Shume (2018) identify two of these competences, collaboration and communication, as major themes in their findings from the ‘Educators in Industry’ programme*.* The theme of communication is further examined by Bowen and Shume (2020), identifying three sub-themes: communication related to collaboration, communication related to problem-solving and the oral presentation of ideas. Communication emerges as a strong outcome for teacher professional learning across all of the nine articles reviewed. Teachers recognised that their placements offered them increased experiences of cultural diversity, and an opportunity to develop the communication skills needed to engage in dialogue with the kinds of people they would not normally meet, in some cases people in other parts of the world (Bowen & Shume, 2020; Gibson, 2013). The *GIFT* programme also places an emphasis on teachers sharing their experiences from the programme, e.g. presenting the outcomes of their summer experiences within their own school, and at local, regional and national-level professional meetings (Barrett & Usselman, 2005). The outcomes reported in the nine articles related to core STEM competences are inextricably linked with the real-world context experienced during the industry placements. Bowen and Shume (2018, 2020) present several examples from teachers’ classroom practices—teachers who have created STEM learning experiences for their students based upon real-world experiences from their placements, and who designed learning opportunities to enhance their students’ own core STEM competences. These will be discussed in the section entitled ‘Teacher Behaviours and Intentions’.

#### Understanding of STEM Roles and Careers

As mentioned in the previous section, Bowen and Shume (2020) describe several, what they call, ‘soft skills’ or ‘twenty-first century skills’ that are ‘necessary for workforce readiness in the current and future global environment’ (p. 75) *and* suggest that teachers are more aware of the skills that their students will need for employability after encountering them first-hand during their industry experience. Participating teachers in all three programmes reported an increased understanding of the range of STEM careers, and the types of work done within certain jobs, particularly engineering. While some pre-service teachers in the UK study (Gibson, 2013) mention gender disparity in engineering roles, it is in a positive light, being pleasantly surprised to see more female engineers than expected and seeing that non-technical staff in the company still have a high level of understanding of the engineering processes. The US-based studies do not reference diversity issues in STEM-related fields of study or employment. In relation to teachers’ own careers, Barrett and Usselman (2005, 2006) are the only authors who report that teachers return to the classroom in September renewed, rejuvenated and with an increased sense of professionalism, which is in line with their stated programme aims, and hence is an aspect of their programme evaluation. Gibson (2013) notes that while some pre-service teachers found the placement confirmed their commitment to a career in teaching, others found it to be an eye-opening experience, challenging them to consider alternative career paths for themselves and imagining themselves working within the industry they had encountered. The *GIFT* programme collects feedback from teachers relating to their intention to participate in further related learning, and intention to think about ways to improve teaching with 70% of the teachers surveyed indicating strong agreement on these items (Barrett & Usselman, 2005).

#### Teacher Emotions

A final set of codes were developed relating to teacher emotions. Thomson and Turner (2019) found significant differences in the emotions reported by teacher participants and after taking part in research lab-based summer programmes, relating to their feelings of excitement, inspiration or confidence. Within the literature reviewed for this study, teacher enjoyment was highlighted in the articles by Gibson (2013) and Barrett and Usselman (2005, 2006); it was not evident in the articles authored by Bowen (2013, 2014, 2015, 2016) or Bowen and Shume (2018, 2020). The only programme dealing with pre-service teachers (Gibson, 2013) was also the only article mentioning ‘apprehension’, noting that in advance of their placements, some teachers expressed concerns that they feel more comfortable interacting with children in school than they expected to feel with other adults in the workplace. Both Bowen (2013) and Gibson (2013) use the term ‘overwhelmed’, in both cases in relation to the sheer scale of industrial operations. Gibson (2013) also refers to a key moment of understanding for one student, who made a link between the individual tasks that students are asked to do in the classroom as part of their design process, and how this really related to what would happen in an industrial context. The light-bulb moment for this student was the understanding that certain steps were more than just something students were asked to do for the sake of a classroom exercise but had actual counterparts in the real world.

Despite these few instances within the literature studied, examples of teacher emotion, or personal revelation, were relatively scarce across the nine publications. Gibson (2013) recognises the transformative potential of industry work experiences for teachers in building teacher identity, but further studies are needed to examine the influence on teachers’ emotions and identity.

### Teacher Behaviours and Intentions

This grouping of codes refers to the influence, or expected influence, in schools and classrooms, of teachers’ STEM industry experiences. Generally, this refers to self-reported behaviour changes, or intention towards behavioural change by teachers, as none of the literature examined contains direct data from learners. While none of the three programmes studied have captured any data directly from students, there are numerous mentions of teachers’ changes (or intentions to make changes) in their classroom practice because of participation in the industry placement programmes. The most common change is in the increased use of lessons that incorporate real-world examples—this includes teachers explaining how something students are learning about is being used, or teachers planning a lesson based around a real context. For example, Bowen and Shume (2018) report on a high-school teacher who had undertaken a placement at a civil engineering firm. Back in the mathematics classroom, this teacher designed an ‘assignment about slope that was set from the perspective of a road construction crew laying storm sewer pipe beneath roadways. In conjunction with this assignment, a guest speaker from the engineering firm gave a class presentation about being a surveyor’ (p. 8). Barrett and Usselman (2005) indicate that 67% of teachers they surveyed strongly agreed with the statement ‘it increased my ability to incorporate “real life” examples of the subject I teach.’ (p. 10.608.12).

Teachers also report on their increased attention to incorporating the engineering design process (EDP) into lessons (Bowen, 2013), and the importance of project-based and hands-on learning experiences in the classroom, was recognised as a ‘critical component’ of learning (Bowen, 2013, p. 23.1134.2). Another example of the use of real-world contexts in the classroom is presented by Bowen and Shume (2018) in which teachers designed STEM lessons to promote the competences needed to increase their students’ employability. They report on cases in which students were ‘cast’ as employees in classroom role-plays, and must be ‘hired’, work in teams, and attend ‘business meetings’ as if in a real-life employment situation. Bowen and Shume (2018, 2020) report an increased commitment from teachers to spend time developing their students’ core STEM competences, particularly communication and collaboration. They note that teachers had a shift in their perception of the importance of students’ communication skills in the classroom, from a minor skill to one worthy of significant attention stating: ‘Nearly all teachers who reflected on the role of communication in problem solving during the summer submitted lesson plans in the fall that provided students with scaffolded communication supports while engaged in problem solving related to engineering design challenges or local community issues’ (Bowen & Shume, 2020, p. 78).

While all the literature reviewed alludes to the importance of developing teachers’ understanding of STEM industries and careers, there is no specific evidence presented in any of the studies regarding increased classroom dialogue about their industry experiences or changed practices in sharing information with students about STEM careers or further studies in STEM.

## Discussion

The goal of this systematic literature review was to uncover the purposes, objectives and outcomes of STEM-learning placements for teachers in industry. This research sought to collate evidence of the influence of such programmes on teachers, and hence student learning, and to inform the design of non-school based placement programmes for teachers. In this section, we discuss the extent to which the findings from this review address our research questions.

This systematic review identified only nine articles related to STEM teacher professional learning in industry. A number of similar programmes have been identified (e.g. ENTHUSE^3^ formerly Teacher Industrial Partners’ Scheme (UK), IgnitED,^4^ Arkansas STRIVE^5^ and Kenan Fellows^6^ (USA), Bridging the Gap^7^ and Teachers for Tomorrow^8^ (Australia)), but these programmes have either not yet published articles, or publications did not satisfy the inclusion criteria of this systematic review, e.g. reports published as grey literature rather than peer-reviewed articles were excluded from this study.

In their systematic review of the literature related to teachers’ perceptions of STEM education, Margot and Kettler (2019) emphasise the demand for extended professional learning opportunities to improve teacher confidence and competence in STEM education. The findings of our systematic review offer insight into the role of industry internships in influencing teacher’s knowledge and understanding of designing STEM learning experiences in their classrooms.

It is interesting to consider the relative merits and challenges of teachers completing a STEM industry placement as part of initial teacher education rather than as an in-service teacher. In the case of programmes for pre-service teachers who will return to study after their placement, the university has a more direct link to the teachers going on placement and the teachers have a commitment to the university. Depending on the programme, a teacher’s performance on their placement may contribute to their final degree award, which may incentivise performance, as is the case in the programme described by Gibson (2013). A university may also have existing internship or work experience programmes which could be leveraged by the programme coordinators or used as a model for recruiting industry hosts for this kind of programme. In the programmes described by Barrett and Usselman (2005, 2006) and Bowen and Shume (2018, 2020), in-service teachers are awarded professional learning credits from the university, thus still utilising the model of awarding performance incentives, even though the credits do not contribute to a degree qualification.

The nine articles reviewed in this study refer to the terms ‘STEM’ and ‘STEM education’, but none of the programmes discussed are reported as being motivated by the need for a more nuanced understanding of what STEM education is, or for increased integration between the four associated disciplines. Gibson (2013) refers to the difficulties that pre-service teachers’ have in conceptualising STEM, stating that: ‘students highlighted individual elements or constituent parts of STEM but evidently found it much more difficult to make connections between and across these’ (p. 305). Bowen and Shume (2020) refer to national policies on STEM education and reference the framework for teaching twenty-first century skills developed by the American Association of College Teacher Education [AACTE] & Partnership for 21st Century Learning [P21] (Greenhill, 2010), but do not explicitly present their definition of STEM education. It is evident that engineering practices and processes are to the fore in the *Educators in Industry* programme (Bowen, 2013, 2014, 2015, 2016), as is the case in the review of Martín-Páez et al. (2019), who find engineering the dominant discipline in 58% of the STEM studies included in their sample. Development of teachers’ core STEM competences (content knowledge combined with communication, critical thinking, problem solving, creativity, collaboration and self-reflection) are identified as clear learning outcomes in each of these programmes, as outlined in Table 2.

An economically focused argument for STEM education emerges in the findings of this review as Bowen and Shume (2020) emphasise the important role of teachers in supporting students to develop future workforce skills, while Gibson (2013) mentions the need for teachers to be ambassadors for a governmental STEM agenda. The programmes reviewed here aim to offer teachers, and by extension, their students, a deeper understanding of STEM and STEM industries in terms of their purposes, processes, contributions and value to society. Gibson (2013) mentions that the school students are ‘more interested’ in hearing about teacher placements in well-known companies or those with a recognisable brand name, perhaps indicating that students do not know much about industrial processes of companies beyond the ones whose marketing campaigns they encounter. The results of this review indicate that spending time with industry personnel discussing their career paths, skills and qualifications is valuable to the teachers, as are tours of facilities and seeing equipment in use. Less evident, however, is the way in which the overall industry experience translates into teachers’ classroom practice and how it informs and shapes their approach to designing STEM learning experiences for their students. Further studies are required to understand this complex process. Teacher educators who design and lead programmes offering immersive STEM industry experiences for teachers must be conscious of making explicit the process of translating learning in the STEM workplace into STEM learning for students in the classroom. The value of this process is evident in the findings of this review—teachers demonstrate a change in their STEM classroom practices when they are required to create a lesson plan or transfer plan as part of the credit-bearing element of their placement experience programme (Barrett & Usselman, 2005, 2006; Bowen & Shume, 2018, 2020).

The literature reviewed here highlights that industry experiences for pre-service and in-service teachers, combined with structured support from teacher educators, can be a powerful approach to both model and develop key competences and pedagogical practices for designing effective integrated STEM learning experiences, across primary and second level education. Such programmes offer the potential to enrich STEM teachers’ professional learning and connections to real-world contexts. Teacher educators can play a crucial role as programme coordinators, evaluators and interlocutors between stakeholders, including industry partners, teachers, students and school communities, funders, policymakers, media and the wider public.

## Conclusions and Recommendations

The three programmes identified in this literature review aimed to support STEM teacher professional learning by providing teachers with opportunities to complete a placement in industry workplaces and gain first-hand experience of STEM roles and careers. The findings from the programmes indicate that participating teachers reported significant personal and professional learning, having developed their core STEM competences and increased their understanding of STEM roles and careers. As a result, many teachers reported increased commitment to providing opportunities for their students to develop their own core STEM competences in the future.

### Recommendations for Teacher Education

Several elements need to be considered in the design, development and facilitation of a programme that enables teachers to complete work experience, internships or externships in industry. Only one study in our sample explores the potential for this type of initiative within initial teacher education, to complement school-based placements for teachers (Gibson, 2013). Given that teacher placement programmes in schools are supported at an institutional level by universities, a robust model for STEM industry placements for teachers may be to embed such an initiative formally within teacher education, perhaps as an alternative practicum experience. Four recommendations for designing and implementing industry placements in STEM teacher education programmes have been identified in this review:

- Develop a common understanding of STEM education between teachers, industry and university.

If programmes are designed to develop teachers’ understanding of STEM education, it is imperative that programme designers articulate their own understanding of STEM education, which has implications on how the programme is designed and implemented. This should form an integral part of the learning experiences for participating teachers and help them to consider how they can apply their workplace learning within a clearly defined understanding of STEM education, thereby translating it effectively to the classroom.

- Support teachers in examining the role of STEM industries within wider societal contexts.

Andrée and Hansson (2020) present a balanced and critical discussion of the possibilities and challenges associated with industry engagement in STEM education. Overall, their evidence points to industry aims being more aligned with ‘preparation for STEM careers, and uncritical attitudes towards science and technology’ than ‘preparing students for critical citizenship and/or activism’ (Andrée & Hansson, 2020, p. 571). They caution about the tensions between different interests and values in education in a democratic society, suggest the need to empower teachers and schools to be critical in negotiating partnerships with the private sector and call for future research to explore what values and norms are being communicated to students as a result of industry influence on STEM education. While the evidence from the literature studied in this review paints a picture of industry workplace learning as a credible pathway for STEM teacher professional learning, it would be beneficial for such programmes to include a critical component to support teachers to examine the role of STEM industries within the wider societal context.

- Collect evidence of the influence of placements on participating teachers and their students.

As stated by Barrett and Usselman (2006), evaluation is a critical component of teacher placement programmes, as both a formative and summative tool. The SWEPT Multi-Site Study (Silverstein & Dubner, 2002) has made a range of validated open-access research instruments available,^9^ which may be adopted for the evaluation of new or emerging programmes.

- Create sustainable funding models and ongoing institutional, industry or government support for programmes.

There are numerous operational and logistical considerations for programme design that are context dependent. The dearth of literature uncovered within this review, and the fact that several programmes that participated in the US-based ‘Scientific Work Experience Programs for Teachers’ (SWEPT) Multi-Site Study between 1998 and 2002 (Silverstein & Dubner, 2002) are no longer operational suggests that programmes such as these are difficult to initiate, manage and maintain over time. Key features for programme design should include strategies for three-way communication between teachers, industry and university.

## Recommendations for Further Research

Further research on the influence of industry placements on STEM teacher professional learning is needed. None of the three programmes examined in this review provided evidence of the influence on students in the primary or second-level classrooms of the participating teachers. Silverstein et al. (2009) present results of a study measuring the impact of the Columbia University Summer Research Program on New York City public high-school science teachers. The authors used a standardised test (New York State Regents science examination) and compared test scores of students of participant and non-participant teachers over 4 years, including the year before the teachers’ participation in the study. They saw no differences in the pass rates of the students of participating and non-participating teachers in the year before joining the summer programme but showed significant increases in pass rates (10% higher) three and four years after participation.

Barrett and Usselman (2006) present an overview of the efforts made towards joined-up evaluation across similar ‘Scientific Work Experience Programs for Teachers’ (SWEPTs) in the USA. A scoping paper (Sloane & Young, 1996) surveyed 35 programme directors and determined six broad areas for evaluation (institutional and programme support, programme implementation, teacher effects, classroom effects, student outcomes, school, and community impact), as well as a consensus on the aims, objectives and challenges facing these programs in terms of the evaluation of student outcomes. A National Science Foundation (NSF)-funded multi-site study of SWEPTs in eight US locations followed, with the final report providing evidence that teacher participation in SWEPT has a significant positive effect on student achievement in science (Silverstein & Dubner, 2002). Barrett and Usselman (2006) propose a ‘SWEPT Theory of Impact’, suggesting that it is possible to maximise teachers’ professional learning through a programme that is structured to provide opportunities to enhance teacher content knowledge, to provide experience of scientific inquiry and to provide effective instruction in best practices such as inquiry- and problem-based learning. Gibson (2013) suggests that the true worth of industry placements may only become evident through a longitudinal study of participating teachers, following their career paths over a prolonged period.

Recommendations for further research studies include:

- Longitudinal observations of teachers to examine their classroom practice and collect evidence of use of their workplace learning. Such studies could also examine long-term career trajectories, including retention in teaching careers, progression to leadership roles, and participation in extracurricular activities related to STEM education.
- Qualitative and quantitative studies with students whose teachers have undertaken STEM industry placements and have later designed innovative learning experiences in the classroom (c.f. Aldous et al., 2019 and Silverstein et al., 2009, for potentially interesting models for controlled sampling).
- Examination of the influence of various industrial host sectors or organisations on participating teachers. Evaluation of the motivations and expectations of the industrial actors (c.f. Andrée & Hansson, 2020), in terms of return on investment (c.f. Silverstein et al., 2009 for a quantitative estimate of the return on investment for Columbia University’s Summer Research Program for Teachers).
- A comparative study on the influence of STEM industry immersion programmes involving both pre- and in-service teachers. Studies included in this review focus solely on pre-service or in-service teachers and the benefit/influence of placements on teachers at different career stages could inform the design and structure of appropriate programmes for each cohort.

As evidenced within this review, whether called internships, externships or placements, time spent by teachers in STEM industries can lead to valuable professional learning which has the potential to influence how STEM learning experiences are designed for students in primary and post-primary classrooms. An effective three-way partnership between education, industry and higher education must take tensions relating to public–private partnerships and the democratic nature of education into consideration and aim to support all parties to address these issues, with a view to empowering the next generation to become critical citizens.

## Footnotes

1 The terms ‘competences’ and ‘competencies’ are both common in recent educational literature; for consistency and coherence, we select the term ‘competences’ in this manuscript, to align with recent publications from the European Commission.

2 https://www.ceismc.gatech.edu/gift

3 www.stem.org.uk/enthuse-partnerships-schools-and-colleges

4 www.igniteducation.org

5 www.ualr.edu/strive

6 www.kenanfellows.org

7 www.bridgingthegap.edu.au

8 www.unisa.edu.au/research/research-projects/teaching-for-tomorrow

9 https://www.scienceteacherprogram.org/SWEPTStudy/instruments.html
